# Exploring utility function in utility management: an evaluating method of library preservation

**DOI:** 10.1186/2193-1801-2-61

**Published:** 2013-02-21

**Authors:** Bin Yan, Feng Shi, Rui-qiang Yu

**Affiliations:** Library, Nanjing University of Posts and Telecommunications, Nanjing, Jiangsu Province 210003 P.R. China; Education College, Shandong Normal University, Jinan, Shandong Province 250014 P.R. China; Economy and Management College, Nanjing University of Posts and Telecommunications, Nanjing, Jiangsu Province 210046 P.R. China

**Keywords:** Book evaluation, Measure model, Utility management, Bellman equation, Library preservation

## Abstract

In order to seek a new method of book evaluation and realize book resources sharing among the regional university libraries, we think that library should collect books of the high utility value in the case of limited funds. We proposed a changing Bellman equation as a utility function and used the explicit functions of the book usage factor and the book usage half-life derived from the utility function as an evaluating method of the collecting books. The results from empirical data given some conclusions such as the varieties diversity, the collected risk, the tendencies of reading varieties, species continuity and so on and a librarian can use the utility management to supplement the collections management.

**Electronic supplementary material:**

The online version of this article (doi:10.1186/2193-1801-2-61) contains supplementary material, which is available to authorized users.

## Introduction

The relationship between supply and demand is undefined in current books from existing editing and publishing. The financing and product prices are becoming competing target among the resources consortia, which result in the cycle and flooding of the phenomena in home market, such as a single kind, vulgar content, lack of language and poor performance etc. Not only are these not able to meet the needs of the multi-level crowds, multi-regional custom, multi-species innovation, multi-fields academic inquiry as the scale, but these also don’t reflect adequately the cultural prosperity and technology in the digital age. However, throughout the book supply chain, it is common that publishing group is to pursue profit maximization and authors are to compile the non-standard with the same materials from different publishers. The single monograph published with depth academic value and low sales is required of high publishing fee self-provided and underwriting these books, so that the domestic book market is hard to find ingenious academic works.

In recent years, library as the main preservation place of literature resources has increased in the number of annual collection with investment increase. But the number of collection of original academic books in Chinese has become less and less. The translation, reprint, repeatedly printing books and leisure books have been full of the library. The developing cultural resources of the network have led a large number of printing books to be in idle. There has been a decline because a lot of readers borrow printing books from the library. The electronic publications have become the most widespread form of publication. A largely open access to electronic journals and free e-book chapters make many readers often use electronic literature resources. That also contributes to the increase of the amount of collection and utilization for electronic literature resources.

Print books are also preferred by readers of students, researchers, children and elders. But as a result of the lack of scientific analogy between print and electronic of books, practical data can not be getting to proof the relationship between reading books and publishing books like academic journals. The results of book evaluation are one-sidedness from “ranking list” of the sales volume of books and are not believable the amount of loan or return books from the traditional methods (Ferne 1993) and statistic formulas of reading books (Yan 2009a, 2009b). Another book evaluation is an uncertainty of artificial interfere factors to manufacturing requirement by an author signing book to sell or a social celebrity reviewing book. Other date of book evaluation is unpredictable immediately with books rewarded by all-leveled governments and society organizations. There are other causes of incomplete data collected from selling and printing books, and little representation because the amount of reading and citation get less. These are unable to form an evaluation standard of all kinds of books.

K. H. Marx (1963) said long ago that every useful article "can be effective in a variety of different ways", so that, "a variety of ways to use article has been found"; "a utility of the article is endowed it with use value"; "use value is only realized in the usage or consumption". Utility, as one of the most commonly used concepts of economics, is a measure of satisfaction which the consumer get when she/he meets oneself demands and desires through consumption or enjoyment, also the capability that goods can meet person’s desires. The utility function represents the function relationship between the numbers of the utility obtained by consumers and the p for consumer goods in consumption. Therefore, the utility in the utility value mainly means the desire of the consumer’s subjective feeling for goods, which the inner strength to meet human needs (Say 1982). More precisely, utility refers to subjective enjoyment or usefulness a person got from consuming of an article or service (Paul and William 1996). The introduction of the utility to manage as utility management can analyse the effective use for the usage property of articles. Their utility contains the composition of the effective use outside the preferences degree of consumers for goods consumed.

The theory is not yet ripe from utility given out a management. The earlier utility management (Zhang and Wang 2005) should have both utility assessment and utility control to guide education as the conscious of the utility management (Xiao 2003), to be an aim of the management by the utility maximization (Li et al. 1998). Utility Management Automation System is a sort of supervisory control and data acquisition system for the electric utility system (Fereidunian et al. 2009). The utility from the economics were applied broadly in finance and insurance about risk and consume such as Markowitz combining model (Feng 2010). Using utility in library was applied to utility function about purchasing books (Lin 2005) and utility modeling of book usage (Zhang 2009) and utility statistics of books (Yan 2009a) and the utility management for the electronic resources (Yan and Xu 2010). The using utility after customer obtaining goods was not involved but the utility were only as the contents of the management. All we mentioned above conveyed a motion of management utility rather than the utility management that had not the utility as the main purpose of the management and also had not established the relation between utility function and applied analysis. It is an issue of the utility management in the article management. We dedicate to using utility management to the possession of articles in the utility value, as the premise of the management with the effective usage of empirical data to analyze the usefulness of continuing degree of these, and to mine the potential usefulness, to make full use of these, the maximum of use impact and management effectiveness (Yan and Wu 2011).

Collecting books in library after being rid of commercial property can express one’s using utility. The utility function is a mathematical tool to express book using utility. We hope to seek a new mathematical measure model as utility function that is one part of the utility management and explore to apply the model to book evaluation. The librarian can use the utility management to supplement the current collection management.

### Literature Review

Utility, we all have known, was proposed by Daniel Bernoulli when he explained St. Petersburg paradox in 1738. Its aim of the theory was to challenge a standard of decision-making with money desire value. In 19th century, early economists, such as William Stanley Jevons, Leon Walra and Alfred Marshall, believed that utility could be measured like the height and weight of people, while John Hicks insisted that under the assumption of ordinal number utility, the utility number was only expressed the order of preference, not the absolute number of utility. After the marginal utility proposed by Jevons, Menger and Walras in early 1970s, it was developed into a value theory by Böhm-Bawerk and Wieser. It is the feature to explain the process of value formation through the subjective psychology. Also, they thought that the value of goods was a kind of feeling and appraisal of people about the utility, which was decided by the utility and rare of goods. Total utility is the total satisfaction that consumers got from the certain consumption of goods and service over a certain period.

### Utility function

If the utility is the satisfactory degree of goods or services for consumer, then utility function means the function relationship in number between utility and the preference of goods. Let the utility function *U* (*x*) as satisfactory model for *x* in the choice set for each to specify degree of preference. Generally *U* (*x*) is between 0 and 1 (Farquhar 1984), which measures satisfactory degree the consumers feel by the consumption of specified combining goods. The utility function, we also have known, has get many mathematic forms such as the power utility function (Jana and Pavol 2010), the exponential utility function (Bhuvaneswari and Seethalakshmi 2011), Epstein-Zin utility function (Tan et al. 2006) etc., or is a cardinal utility function and a ordinal utility function. And the utility function has also been applied to many fields such as the information utility function, the multi-attribute utility function, the rent-seeking utility function, the traffic utility function, the objective utility function, the cost utility function, the HARA (hyperbolic absolute risk aversion, absolute risk aversion) utility function, the PD (partial distribution, partial tail distribution) utility function, the PRA (power risk aversion) utility function, the Logit (logistic) utility function and so on. These functions were defined from implicit function hypothesized to explicit function applied.

Assuming that the letter *n* is types of goods consumed and symbol *x* is consumption vector, and the utility function is the *U*(*x*) = *F*(*x*_*1*_, *x*_*2*_, ⋯ , *x*_*n*_). The function is not an empty set, owing to goods consumed actually, and the preference is monotonic relationship. In the real domain of Euclidean space, the $U:R_{+}^{n}\to R$, the *R* is mapping every point *U* in the real fields $R_{+}^{n}$. The existence of the utility function has been demonstrated (Zeng et al. 2006) (Chen et al. 1999) that it is monotone, continuous, differentiated. Several common binary explicit utility functions are as follows.

1. The alternative (linear) utility function: *u*(*x*_1_, *x*_2_) = *a*_1_*x*_1_ + *a*_2_*x*_2_
2. The complementary utility function: *u*(*x*_1_, *x*_2_) = min (*a*_1_*x*_1_ + *a*_2_*x*_2_)
3. The merging utility function: *u*(*x*_1_, *x*_2_) = *u*(*x*_1_) + *u*(*x*_2_)
4. The alternative (exponential) utility function: *u*(*x*_1_, *x*_2_) = *x*^*a*^_1_ + *x*^*a*^_2_
5. Cobb - Douglas utility function: *u*(*x*_1_, *x*_2_) = *x*^*a*^_1_ • *x*^a^_2_

When the total utility in the application uses multiplication rule, if any element is zero, the total utility value is zero. When it uses addition rule, if every element value in the total utility is independent, then each other can be linear compensated.

Cobb - Douglas utility function:

1 $$U\left(x\right)=x_{1}^{a_{1}}\cdots x_{n}^{a_{n}}=\prod_{i=1}^{n}x_{i}^{a_{i}}\;\left(a_{i}\geq 0,\;\sum a_{i}=1\right)$$

Here (1) the *U* (*x*) is the utility, and the *x* = (*x*_1_, *x*_2_, ⋯ , *x*_*n*_) is a vector of goods consumed. The utility consumer got is power product of all kinds of goods amount.

The utility theory we known from economics was established according to the principle of diminishing marginal utility and total utility maximization. Consumers buy goods in order to obtain the utility and they are willing to paying more for big marginal utility of goods. It means that consumers take a standard of the marginal utility as to pay for goods. The utility, in the same way, can be obtained from the different combinations of several articles. Some modern economists think that it is not necessary for consumers to feel the satisfactory degree of precise measurement, only know consumer’s utility order through their preference for different commodities. All of these utility functions are presented many economic fields in the finance, the stock market or the insurance and are only not applied to an article or a book.

### Bellman equation

The general function of the utility to resolve a problem can be divided into optimization and control, expectation utility, preferences and risk, substitution and so on. Its function makes up elements of the value of the goods such as amount and increment as variable with the array, and parameter as variable coefficient with normalization. There are commodity, time and price in variable as unconstraint or constraint condition. The unconstraint condition is only to establish a utility function relationship and the constraint condition is to solve the function value. In the constraint condition the cost total is much application as variable condition. There are also many variables such as the utility total (*U*), scalar quantity (*X*), the number of similar products or the amount of costumer (*x*_*n*_), price (*p*_*n*_) and the constraint condition such as the total funds (*w*) and total time (*T*). They are established according to different application to the specific event in the utility function.

Bellman equation is often used of the multi-stage decision-making under uncertainty modeling. It was original as the engineering control theory in applied mathematics, and then became an important tool in economics theory that mainly solved the problem of optimal control theory within any time. It usually refers to the dynamic programming equation and associates with the discrete-time optimization (2) (See Wiki Bellman equation 2011a).

2 $$\begin{array}{l} V\left(x_{0}\right)=\max_{{\left\{a_{t}\right\}}_{t=0}^{\infty }}\sum_{t=0}^{\infty }\beta^{t}F\left(x_{t},a_{t}\right)\\ s.t.\;a_{t}\in \Gamma \left(x_{t}\right),\;x_{t+1}=T\left(x_{t,}a_{t}\right),\;\forall t=0,1,2,\cdots \end{array}$$

Notice that *V*(*x*_*0*_) notation has been defined to represent the optimal value that can be obtained by maximizing this objective function subject to the assumed constraints. This function is the value function. It is a function of the initial state variable *x*_*0*_, since the best value obtained depends on the initial situation.

Bellman equation is proposed the optimality principle to separate the current decision-making from the future decision-making. The stage and the state in the first term must constitute the optimal decision in the future, which reflects the basic idea of the dynamic programming method.

The continuous time optimization should require to partial differential equation, usually with the Hamilton-Jacobi-Bellman (HJB) equation (3) (See Wiki Constant elasticity of substitution 2011b).

3 $$V\left(x\left(0\right),0\right)=\min_{u}\left\{\int_{0}^{T}C\left[x\left(t\right),u\left(t\right)\right]\mathrm{dt}+D\left[x\left(T\right)\right]\right\}$$

The *C[−−]* is the scalar cost rate function and *D[−−]* is a function that gives the economic value or utility at the final state, *x*(*t*) is the system state vector, *x*(*0*) is assumed given, and *u*(*t*) for *0* ≤ *t* ≤ *T* is the control vector that we are trying to find.

The Constant Elasticity of Substitution (CES) is a property of some production function and utility function. It is a type of production function that displays constant elasticity of substitution. In other words, the production technology has a constant percentage change in factor (e.g. labor and capital) proportions due to a percentage change in marginal rate of technical substitution. The two factors (Capital, Labor) CES production function introduced by Solow and later made popular by Arrow, Chenery, Minhas, and Solow is equation (4):

4 $$Q=F\cdot {\left(a\cdot K^{r}+\left(1-a\right)\cdot L^{r}\right)}^{\frac{1}{T}}$$

The *Q* is the output, the *F* is the factor productivity, the *a* is the share parameter, the *K* and the *L* are the primary production factors (capital and labor), the *r* is the elasticity of substitution (See Wiki Hamilton–Jacobi–Bellman equation 2011c).

When two variables become the proportion of the relative charges, there is an elasticity relation between variables. The CES has the nature of the production and utility function and means a specific type of aggregation function to combine two or more types of consumption, or a function relation among two or more species productive inputs into overall amount. And the CES utility function means the relation between the consumption amount and the utility for consumers of the elasticity of substitutive inconvenience.

Bellmen equation was applied to the resources allocation (Zhao 2004), assuming the *A* is the total number of some kind of economic resources, and assign it to the *N* (*N* ≥2) economic units *U*_*i*_(*i* = 1, 2, ⋯ , *N*), assigned *i* to the amount of resources of economic units *U*_*i*_ is the *A*_*i*_ (*A*_*i*_≥0), then the revenue *L*_*i*_ of the economic units *U*_*i*_ is the *L*_*i*_ (*A*_*i*_). If the maximum total Revenues ${\tilde{L}}_{N}$ (*A*) are earned by economic resources *A*, the resources allocation will earn the maximum total revenues as follow equation (5):

5 $${\tilde{L}}_{N}\left(A\right)=\max_{\sum_{i=1}^{N}A_{i}=A}\left[L_{1}\left(A_{1}\right)+L_{2}\left(A_{2}\right)+\cdots +L_{N}\left(A_{N}\right)\right]$$

The most optimal allocation of total resources is as follow equation (6):

6 $${\tilde{L}}_{N}\left(A\right)=\max_{0\leq A_{N}\leq A}\left[L_{N}\left(A_{N}\right)+\max_{\sum_{i=1}^{N}A_{i}=A}\left\{L_{N-1}\left(A_{N-1}\right)+\cdots +L_{1}\left(A_{1}\right)\right\}\right]$$

The project evaluation in the venture capital was established mathematical model (6) with Bellmen equation (Qian and Huang 2003), where the *F* is a value of investment option, the *x*_*t*_ is a current state variable for enterprise, the π is a profit function for an enterprise, the *r* is risk-free profits, the *V* is value to convert cash flows in the future for the project, the *α* is a expected growth rate of the cash flows for the project, the *σ* is a fluctuating rate of the cash flows for the project, the *t* is current time, the *T* is a expiration date of the investment option, and the *I* is investment costs. When an enterprise selects decision-making *u*_*t*_ at the length of time each stage *Δt*, the discount factor is 1/(1+*rΔt*), the enterprise has the maximal expected value with the current income and the future investment option (7).

7 $$F_{t}\left(x_{t}\right)=\max\left\{\pi_{t}\left(x,u_{t},t\right)\mathrm{\Delta t}+\frac{1}{1+\mathrm{r\Delta t}}E_{t}\left[F_{t+1}\left(x^{'},t+\mathrm{\Delta t}\right)x,u\right]\right\}$$

When an asymmetric monopolistic competition model (8) was established with utility function, a set of different brands in the same type of products (*x*_1_, *x*_2_, ⋯ , *x*_*n*_), the substitution parameter *ρ* between 0 and 1, it reflected the strength or weakness for diversity preferences of consumers (Weng and Chen 2006).

8 $$U=u\left[x_{0},(\sum_{i}{X_{i}^{\rho })}^{\frac{1}{\rho }}\right]$$

The early utility made a point of the economics such as consumption and the utility function was applied to the many economical fields. The current utility in our research is to use articles such as reading books in library collection. These utility functions are not adapted to the evaluation as a method and exploring new utility function is the need in order to evaluate books collected.

### Methodology

The basic concepts of the utility management based on various expositions above all can be extracted from the utility (Say 1982) and the utility value (Zheng 2003) (Yu and Hu 2007) and the utility function (Zhang and Chen 2000) (Koksalan and Ozpeynirc 2009) and the utility theory (Farquhar 1984) (Dong 2007) as a management theory. It can be summarized mainly as follow: (1) the utility of resources or the product as a unit of measure; (2) the amount of using resources or the using product as evaluation; (3) determining the user’s preferences through the measure values; (4) the new resource allocation or the article diversity to hedge against the risk through the measure values; (5) continuous time utility assessment. Its goal is to mine the potential usefulness of the article and to predict the degree of the article continual used and to reach the article full used and maximal effects.

Because book evaluation is one of important work in library management, utility function may be applied to the evaluation as a major method and become one part of utility management. Bellman equation in utility functions has the property of the multi-stage decision-making under uncertainty modeling and very adapts evaluating to collecting books and using books as a method in library management.

While the utility of the article in the utility management is a use role, the total utility can be separated into a basic utility and factors utility. The basic utility is an inherent use value of the article and it may get a theoretical use role through the market assessment. The factors utility is a real use role of the article and it is uncertainty caused by various factors. If an article was not properly managed, it might cause the total utility to reduce. Otherwise if it was maintenance well, its total utility would extend or increase. The total utility may predict a concrete article- how many to reduce or extend on the basis of actual usage data. Because each library has its own structure of library preservation, different characters of each librarian make books existed utility as soon as collected after consumed and had not presented consumed utility for the library. The books in library preservation have the basic utility such as knowledge, appreciation, conservation exhibit etc. and other utility as factors utility will present use data in loan, read, citation estimation and other. The utility management shows all practical use roles in total utility and can have a kind of book to play a different utility part in every region. On these grounds we become Bellmen equation into follow (9) as a new mathematical modeling of utility function for book evaluation:

9 $$\begin{array}{ll} U\left(x\right)= & \max_{0<t\leq \infty }\left[U\left(x_{0},y_{0},t\right)\right]\\ +\max_{1\leq t_{e}\leq T}\left\{\sum_{j=1}^{n}\mathrm{\beta U}\left[x_{j}^{r}\left(t_{e}\right),y_{j}\left(t\right),t\right]\right\} \end{array}$$

Here the *U*(*x*) is the value of optimal utility in formula (9); it is divided into the basic utility *U* (*x*_*0*,_*y*_*0*_) at the first term on right side of formula and the factors utility $U(x_{j}^{r},y_{j})$ at the second term on right side of formula. The basic utility of books had collected utility soon as preserved by library is reflected in books of the inherent value and heritage value of literature. The collected time *t*=*t*_*e*_ is determined the state (*x*_*0*_, *y*_*0*_) of kinds of books which is a continuous function of time, that is, the longer is time, the greater is the basic utility. The factors utility of books is uncertain, and $x_{j}^{r}$ is variables of variety library preservation, where the *r* is the constant of substitution elasticity, the β<1 is parameters converted or discount factor with use effect by readers. When time *t*_*e*_ and total preservation *x* are certain value, *x*_*j*_ is preservation variable at every preservation place, *y*_*j*_ is use variable of readers, they are a function of discrete random variables. The most optimal factors utility is to require the utility function in utility management. The factors utility may divide into collection-related utility such as borrowing, download within the network, guided reading, recommendation, communication, display and so on, and utility of nothing to do with the collection such as references, reviews, awards and so on. If *x*= (*x*_*1*_, *x*_*2*_, ---, *x*_*n*_)^*’*^ is vectors of the preservation, |*A*| is a count matrix of kinds of the preservation and if *y*=(*y*_*1*_, y_2_, ---, y_n_) is vectors of the usage, |*B*| is a count matrix of kinds of the usage.

In utility management amount of book utility is set *x*= (*x*_*1*_, *x*_*2*_, ---, *x*_*n*_)*’* when *t*=*t*_*e*_, then *y*= (*y*_*1*_, y_2_, ---, *y*_*n*_) is all use vector when collected time *t*=*t*_*e*_, utility time *T*≥*t*_*e*_ is use time of readers. The factors utility of books $U=U(x_{j}^{r},y_{j},t)$ when the *r* is constant, the *y*_*j*_ = *y*_*j*_(*x*, *T*), *U* = *U*⌊*y*_*j*_(*T*), *t*⌋. The factors utility predicted in the theory should have a fixed value *U*_*0*_, and the utility management will present how to abridge or prolong theory utility *U*_*0*_. Utility different is *ΔU* = |*U*_0_(*x*, *y*_0_, *T*) − *U*(*x*_*j*_, *y*_*j*_, *t*)| ≤ *ε* according to management effect. When *t*=*t*_*e*_ and *x*= ∑*(ax*_*j*_), *t*_*e*_<*t*≤*T* and actual count of usage is *y*_*j*_ (*x*), and *y*_*0*_ is theory count of usage. When *y*_*0*_≠*y*_*j*_, then 0<|*y*_*j*_- *y*_*0*_|<δ is existence. Assuming that *S*(*y*_*j*_) is a use function, theory use function *S*(*y*_*0*_)=*A*, |*S*(*y*_*0*_)-*A*|<ε, then $\lim_{y_{j}\to y_{0}}S\left(y_{j}\right)=A$. Same $\lim_{y_{j}\to y_{0}}U\left(y_{j}\right)=U\left(y_{0}\right)$ exist in the extreme value of the factors utility.

We can define some variables and derive some explicit functions from in (9). The methods of book evaluation are as follows:

- At the right second term of the formula (9) if the value of total factors utility is less than or equal to 1, the discount factor *β* is effective, and if it takes an arbitrary plurality, the discount factor *β* is ineffective. The discount factor as a utility weight can be artificially set or can also be obtained through use vector.
- At the right second term of the formula (9) the multivariate of the total use is simplified to mono variable that makes up function with time *t*, the amount of usage is not direct proportion to the amount of the preservation. Assuming it to be an exponential relationship:
  11 $$y\left(x,t\right)=\left(Ae^{-\mathrm{\lambda x}}+a\right)t\;\left(A>0,\;\lambda >0\right)$$
- When the formula (11) is done the partial derivatives with time *t*, and we get the extreme value *Ae*^− *λx*^ + *a* = 0, *x* = − ln(−*a*/*A*)/*λ* that attain the maximum use of the preservation. Here the amount of the preservation is *x* = (*x*_1_, *x*_2_, ---, *x*_*n*_), the *n* is places of the preservation, the *λ* is species, the *x* is a copy, and *λx* is the total copies of every species.
- After the right second term of the formula (9) is simplified, assuming the use variable is an amount of lend books or holding days of books, the utility function *U*=*U*(*y*, *t*) is done the partial derivatives with time *t*, and we get an amount of loan books or holding days of books at every piece of time. If we need to solve the utility of different kinds of books, it is the ordinal utility that is the first, the second, the third, etc. to reflect the utility ordinal or grade. It is also to rank order method according to reader preference, that is, usually to use ranking list method.
- After the right second term of the formula (9) is done the partial derivatives with *U*(*x*_*j*_), we obtain $\frac{\partial U\left(x\right)}{\partial U\left(x_{j}\right)}=\frac{1}{\beta }$, in which discount factor is an actual utility factor, it is the book usage factor (BUF) (Yan 2009b), and can be obtained by empirical data.
  12 $$\begin{array}{ll} \mathrm{BUF} & =\frac{\partial U\left(x_{j}\right)}{\partial U\left(x\right)}\\ =\frac{\begin{array}{cccccc} \mathrm{checkout} & \mathrm{number} & \mathrm{of} & \mathrm{one} & \mathrm{kind} & \mathrm{books} \end{array}}{\begin{array}{ccccccc} \mathrm{total} & \mathrm{checkout} & \mathrm{number} & \mathrm{of} & \mathrm{same} & \mathrm{class} & \mathrm{books} \end{array}} \end{array}$$
- At the right second term of the formula (9) the multivariate of the total use is simplified to mono variable that makes up function with time *t*, book usage half-life (Yan and Wu 2011), is illustrated as (13):
  13 $$r=\frac{\mathrm{dx}}{\mathrm{dt}}=kx^{n},\;\mathrm{dt}=-\frac{\mathrm{dx}}{kx^{n}}$$
- When the *n*=1, *t* = 0, the total amounts of checkout books is *x*_*0*_, *c*=ln *x*_*0*_, see (14):
  14 $$\ln x-\ln x_{0}=-\mathrm{kt},\;\ln\frac{x}{x_{0}}=-\mathrm{kt},\;x=x_{0}e^{-\mathrm{kt}}\;T_{\frac{1}{2}}=\frac{\ln2}{k}$$
- A principle of the utility maximization leads to exist in a possibility of the greatest use risk. The individual behavior to judge, that is a decision-making, is in order to obtain the maximum value of the expected utility, so called the preference, and not necessarily to gain the maximum benefits. If some use amount *y* and satisfaction degree *u* form utility function *u*=*u*(*y*), it has the first derivative equal to zero and the second derivative be less than zero. When the maximum total utility is unchanged, some utility can exist in the use risk because every use variable consisted of every kind of utility which some small utility was ignored.
- The substitution role exists in the cardinal utility, with the *r* denoted a coefficient of elasticity of substitution, and the *r* is obtained $r=\frac{\mathrm{\Delta y}}{y}/\frac{\mathrm{\Delta x}}{x}$ the *y* and *x* denote different kind of preservation and ∆*y* and ∆*x* denote different kind of use preservation.

## Results

The data collection was from information management system of some university libraries and statistical results were also from the methods of the changing Bellman equation. We can get some aspects as follows.

### Diversity judgment

It is an inevitable result of the natural development that the article diversity is the same as the biological diversity. The biological diversity is environmental needs for maintaining ecological balance and sustainable development, while the article diversity is the guarantee of human and environment for harmonious living together in a long period. The category diversity in library preservation can meet the needs of readers’ choice, and judgment of the diversity could obtain through book usage factor calculated. We calculated some book usage factors about the management as keyword in library of China University of Mining and Technology (CUMT). See Table 1.

**Table 1:** **Different BUFs of management’s books in library of CUMT**

Title	CLC number	BUF	Checkout times	Checkout times of same class
University management	G627	1.000000	5	5
“Management of National Economy” Self-counseling	F2-42	1.000000	1	1
Pharmacy Management	R95	1.000000	3	3
Western Management Famous Summary	C93-7	0.800000	48	60
Modern Management of the Coal Enterprises	F407.216.1	0.562500	9	16
Coal Industry Production Management	F407.162	0.454545	5	11
Security Management Principles	X9-05	0.389831	23	59
Resources Management	F205	0.351351	13	37
Modern Book Industry Enterprise Management	G231	0.272727	3	11
Education Management	G46	0.262626	29	110
Archives Management	G271	0.258741	37	143

The utility management can express article diversity and their diversity using as total utility in utility function. Once data collected were calculated to ignore the smallest classes of utility, books exist in diversity short. The value of BUF in Table 1 is greater than or equal to 0.33 such as CLC (Chinese Library Classification) number G627, F2-42, R95, C93-7, F407.162.1, F407.162, X9-05 and F205 to judge single class or diversity short. We think the value of BUF should be smaller than or equal to 0.3 because of the article diversity at least 3 kinds.

### Early warning for risk

We get the total amount from certain number of users using about different species in same class of articles in a certain period of time and call as all the utility *U*. The relationship is between a certain use time *T* and total utility *U* as the article time *T*=*t*_*n*_-*t*_*0*_+1, to establish numbers of usage *X* and time *t* with a function *X*=*X* (*t*), then the area covered is total utility. However, the different purpose of articles may have different numbers of usage, which occupy different weighty. Use weighty *a*_*i*_ of different purpose is first obtained, and then we can get the total utility *U*(*x*) = *U*(*x*_11_, *x*_22_, ---, *x*_nm_).

15 $$\begin{array}{ll} U\left(x_{1},x_{2}\cdots x_{n},K,T\right)= & \sum_{i=1}^{n}a_{i}\sum_{j=1}^{m}{\left(\mathrm{kxt}\right)}_{j}\left(n\leq m\right),\\ \sum k_{i}=K,\sum a_{i}=1 \end{array}$$

Here *K* is amount of copies every kind. One of use weights becomes a_1j_>>a_(n-1)j_, while *U*(*x*)≈a_11_*x*_11_+a_12_*x*_12_+---+a_1m_*x*_1m_. Use duration is likely to the sum of all use time after articles into the management. This usage is determined by number of articles or use time. We know that use of book includes reading, citation, review, collection etc., data of which collected also have each different and the reading book (loan) is only main use or the greatest than all other use.

We have get the top ten kinds of books about economics and management on the ranking list of the sales from “*China Publishers*” (2010) and these books were collected at some university library and show Table 2.

**Table 2:** **Books collected about economics and management at some university libraries**

No.	Title	Publish date	Library name	Citation times
1	The World is Flat	2008.7	(1)(2)(3)(4)(5)(6)(d)	1734
2	Lang Xian-ping Said: the New Imperialism in China	2010.1	(1)(2)(3)(4)(5)(6)(d)	8
3	Currency War	2007.6	(1)(2)(3)(4)(5)(6)(d)	203
4	Currency War 2	2009.7	(1)(2)(3)(5)(6)(d)	10
5	Lang Xian-ping Said: the New Imperialism in China 2	2010.5	(1)(2)(3)(5)(6)(d)	
6	Detail Determining Success or Failure	2004.2	(1)(2)((4)(5)(6)(d)	690
7	Future Enterprise’ Road	2010.4	(1)(2)(4)(5)(6)(d)	1
8	Lang Xian-ping Said: Who is to Save China’s Economy	2009.9	(2)(3)(5)(d)	
9	The Power of the Company	2010.8	(1)(2)(3)(6)(d)	5
10	Lang Xian-ping Said: Why is it so Hard Our Days	2010.9	(1)(3)(6)	

Note: *the (1) denotes library of People’s University of China; the (2) denotes library of Central University of Finance and Economics; the (3) denotes library of Nanjing University; the (4) denotes library of Southeast University; the (5) denotes library of Nanjing University of Posts and Telecommunications (NUPT); the (6) denotes library of Nanjing University of Finance and Economics; the (d) denotes Duxiu Scholar of ChaoXing company in China.

*Statistical date: February 25, 2011.

*Citation of statistical date: March 9, 2011.

In Table 2 the top books were not collected by all university libraries on the ranking list of the sales. There were 70 copies of every kind at some book store in Nanjing. Because data of citation from CNKI are all from the kind of book, data of reading are likely one library or some libraries at the region area.

**Figure 1:**
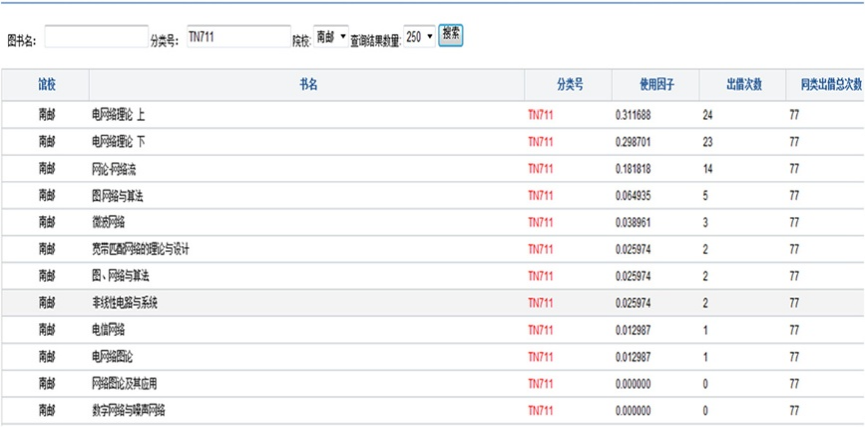
**Part retrieval results of books about CLC number TN711.**

There were 51 kinds of books collected by NUPT about CLC number TN711, network, by the end of 2009 and total valid lending times were 77. The part retrieval results show fellow Figure 1 by our program designed and the top five kinds of books are follow as Table 3.

**Table 3:** **Contrast BUFs among different kinds in same class of books**

Title	BUF	Checkout times	Total checkout times
Electrical Network Theory (Volume one)	0.311688	24	77
Electrical Network Theory (Volume two)	0.298701	23	77
Net Theory - Network Flow	0.181818	14	77
Diagrams, Network and Algorithms	0.064935	5	77
Microwave Network	0.038961	3	77

Table 3 shows the first five kinds of books and 69 times of lending, occupying 90% of total times. Other books were used less and even neglected to use. The books, they mostly use, are a few of kinds and are published in recent 3–5 years. It is uncertain at book supple chain from publish, sell, collection and read.

The library preservation has existed in the utility and the risk together. The presence of risk is often a potential element that undermines utility balance. There are two extreme aspects of very high and very low or never use amount. The high usage will be asked to continue to increase collections and to result in deformity collection phenomenon of additional a few kinds and many copies of one kind. The books, they used less or never used, keep in purchasing as a simple collection utility with no value of BUF. Books as collection may refer to high value of BUF in some university libraries. Our common goal will effectively configure and share with literature resources in regional area that commonly use books would collect in every university library and books of feature categories of profession or specialty would collect completely in different university library formed special collection.

### Preference continuing

In many practical applications, the utility function can get an explicit function of multiple high-order equation group and extreme solution. For example in book usage half-life (BUHL), according to book classification, books belong to different classes, there are different kinds in the same class, and the same kind may has number of checkout times or checkout times, taking-out days, appointment times, citation times, book reviews, number of prints, number of editions and so on. If taking a category of books and checkout amount, according to defining BUHL, we take annual average *x*_*0*_ of checkout books within three years that were same class into library. When the number of checkouts reduces to a half of the annual average, this year would be a half-life of using books, meaning a degree of aging books.

**Figure 2:**
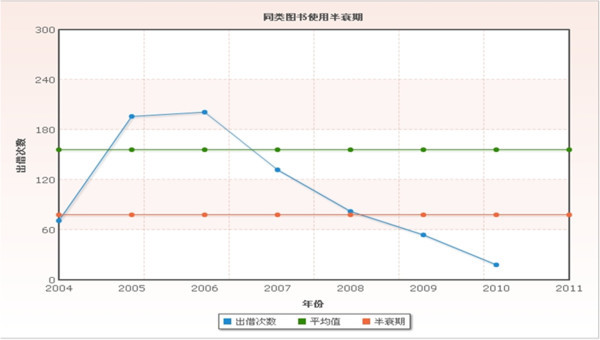
**Book usage half-life of CLC number R94.**

There were, in the same time, 284 kinds and 1101 copies with CLC number R94, pharmacy, in library of China Pharmaceutical University in 2004. Book usage half-lives of the classification up to April 2010 are as follows Table 4 and Figure 2.

**Table 4:** **Book usage half-life of CLC number R94**

Year	2004	2005	2006	2007	2008	2009	2010
Checkout	71	196	201	132	82	54	18

**Figure 3:**
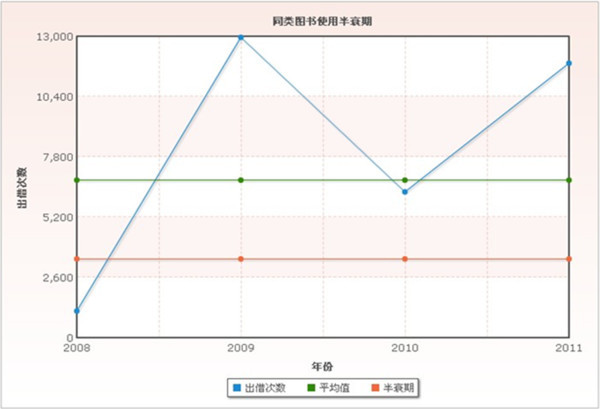
**Book usage half-life of CLC number TP.**

When BUHL shows a peak again in 2011, the number TP of CLC exists in continual preference of readers. See Figure 3.

### Substitution role

Such as print books and electronic books in preservation resources exist in amount of different use and collection in different period, and the amount of use and collection print books was far greater than its e-books in the last century. But now the rate of print books between the amount of use and collection is quite some even lower than one of the e-books. In the future the electronic resources will be flooding in published market but re-use of print books will improve.

In library of NUPT, there were 800 thousand copies numbers of total library preservation and 506071 times of total checkout in 2005. There were 1 million copies numbers of total library preservation and 642622 times of total checkout in 2007. There were 19 literature database in Chinese and English and 224148 download pieces in 2005 and 37 literature database in Chinese and English and 149813 download pieces in 2007. Assuming that the letter *y* denotes print books and the letter *x* denotes electronic literatures. The constant of coefficient of elasticity of substitution *r* is follow:

$$\begin{array}{ll} r_{2005} & =\frac{\mathrm{\Delta y}}{y}/\frac{\mathrm{\Delta x}}{x}\\ =\frac{506071}{8000000}/\frac{224148}{19}\approx 0.6326/11797\approx 5.362\times 10^{-5} \end{array}$$

$$\begin{array}{ll} r_{2007} & =\frac{\mathrm{\Delta y}}{y}/\frac{\mathrm{\Delta x}}{x}\\ =\frac{642622}{10000000}/\frac{149813}{37}\approx 0.6426/4049\approx 1.587\times 10^{-4} \end{array}$$

When *r*_2005_<*r*_2007_, it shown that print books in 2005 was less checkout numbers than in 2007, but reading print book in 2005 was more weight than downloading electronic literatures as compared with in 2007. The electronic literatures from 2005 to 2007 had not substituted the print books.

### Discussion and Conclusions

The utility is no longer a preference for obtaining commodities but the preference for articles using. It means that a user was enthusiastic about the use of some kind of article. If the long-time or a lot of certain articles could be used, the user who loses interest would attempt to try new commodity and substitute for using article, and the amount of use would decline and total utility would have the maximum value. According to the definition of marginal utility to satisfy the final consumer commodity utility of that unit, so called total utility increment, there is the marginal utility for use of the article. It says that whether it is commodities or articles, its commodity property has been lost, only property of use remains. The utility role can be expressed by utility value and utility function. Only commodities are the economic utility from the view of consumer and articles are the management utility from the view of user.

With the amount of book publishing and its marked price have increased year by year, the library is difficult to maintain its previous method of procurement with the book procuring fund increased slightly. Also due to the collection in the form of electronic publication, nearly half of the literature procuring fund spends the literature databases to bring about same collection among university libraries. The utility in library management is a role of using book so we propose the utility management as a complement of the collection management in library. We hope to know whether readers read collected books or not through analysis results and make some suggestions.

Many early methods of book evaluation lack science and system unlike the journal impact factor. Many utility functions were defined as implicit function and applied to economics as the power function form of the logarithm and the exponential function of explicit function. There is not a utility function for book evaluation in all of these. The study has found Bellman equation can be required on the evaluation overall quality of books as measure model in library management. In our exploration we introduce the possibility of book evaluation utility function on the basis of library collections. We propose the book usage factor and the book usage half-life from changing Bellman equation as an evaluation method and to improve a method of traditional evaluation and assessment of books. A set of assessment methods based on utility management is able practically to evaluate the collecting books and forecast readers to obtain the knowledge and skills preferences.

If the utility is a degree of satisfaction for consumer to buy goods, the utility will be a degree of satisfaction for user to use amount of articles by managers. The mathematical modeling for the utility management needs to establish a functional relationship between articles managed and amount of articles used and is able to give the maximal utility through analyzing management factors. Users may give different results to articles used and all results will give social utility and economic utility. Then utility management is to evaluate the synthetic utility that issues from articles managed after these used. The kind of evaluation takes a method about management factors for utility and it will predict the possibility to give maximal utility with a variety of factors. From evaluating books we have got that:

1. Being clear the use value of the articles in utility;
2. Determining the changing Bellman equation as a utility function in utility management;
3. The book usage factor and the book usage half-life are an explicit function in the utility function;
4. Two explicit functions play a better role in the book evaluation of collection;
5. Utility management as collection management supplement.

The utility management applied to books as an attempt and it can bridge the gap between the library and book supply and also provide for a measure model to improve a method of traditional evaluation and assessment of books. It will provide an optimal path for the preservation of cultural resources and made full use of the current resources. The study on utility evaluation for books based on the theory of utility value contains designing the standard formula, which converted lending books to the factor of statistical data, and book usage factor for evaluation method for book utility, which judged utility value of the kind of book for reader to obtain knowledge, and book usage half-life, which predicted readers reading trend for some class of books, and the elasticity coefficient of substitution, which established early warning of using fluctuations between print books and e-books. All mathematical formulas above need a great amount of data to illustrate reliability and give quantitative evaluation. The book evaluation needs to be further research by a utility function like the coefficient of elasticity of substitution in the future.

Total utility sums to all various utility for one kind of book or one class of books, while derivative utility does not direct become part utility and is only to judge its maximum utility. The statistical results display that “ranking list” could not affect library collection but the risk was a lot of collected books being in idle. Many kinds of books lack of the diversity and are not satisfaction for reader need. A kind of book is not read in a university library but has many readers in other university library. This needs to build special library collection among regional university libraries and make books in idle be maximum utility. It is feasible of the evaluation books with the changing Bellman equation and its results proved that the varieties diversity of books collection could meet the needs of readers and achieve book species continuity. While there was risk of collecting books that were not used for a long time, the kinds of books could be used in other university library. These university libraries may consider regional resources sharing. We proposed a changing Bellman equation as utility function and some results of the evaluation books were from its derivative function. However some results were lack of more data analysis like the constant of coefficient of elasticity of substitution because of more electronic literatures download in current now, so as above all are just as a reference method.

## Authors’ original submitted files for images

Below are the links to the authors’ original submitted files for images. Authors’ original file for figure 1 Authors’ original file for figure 2 Authors’ original file for figure 3
