# Supplementary figures and images for: Exploring utility function in utility management: an evaluating method of library preservation
Source: Springerplus. 2013 Feb 21;2(1):61. doi: 10.1186/2193-1801-2-61 (PMC3627857; doi:10.1186/2193-1801-2-61)

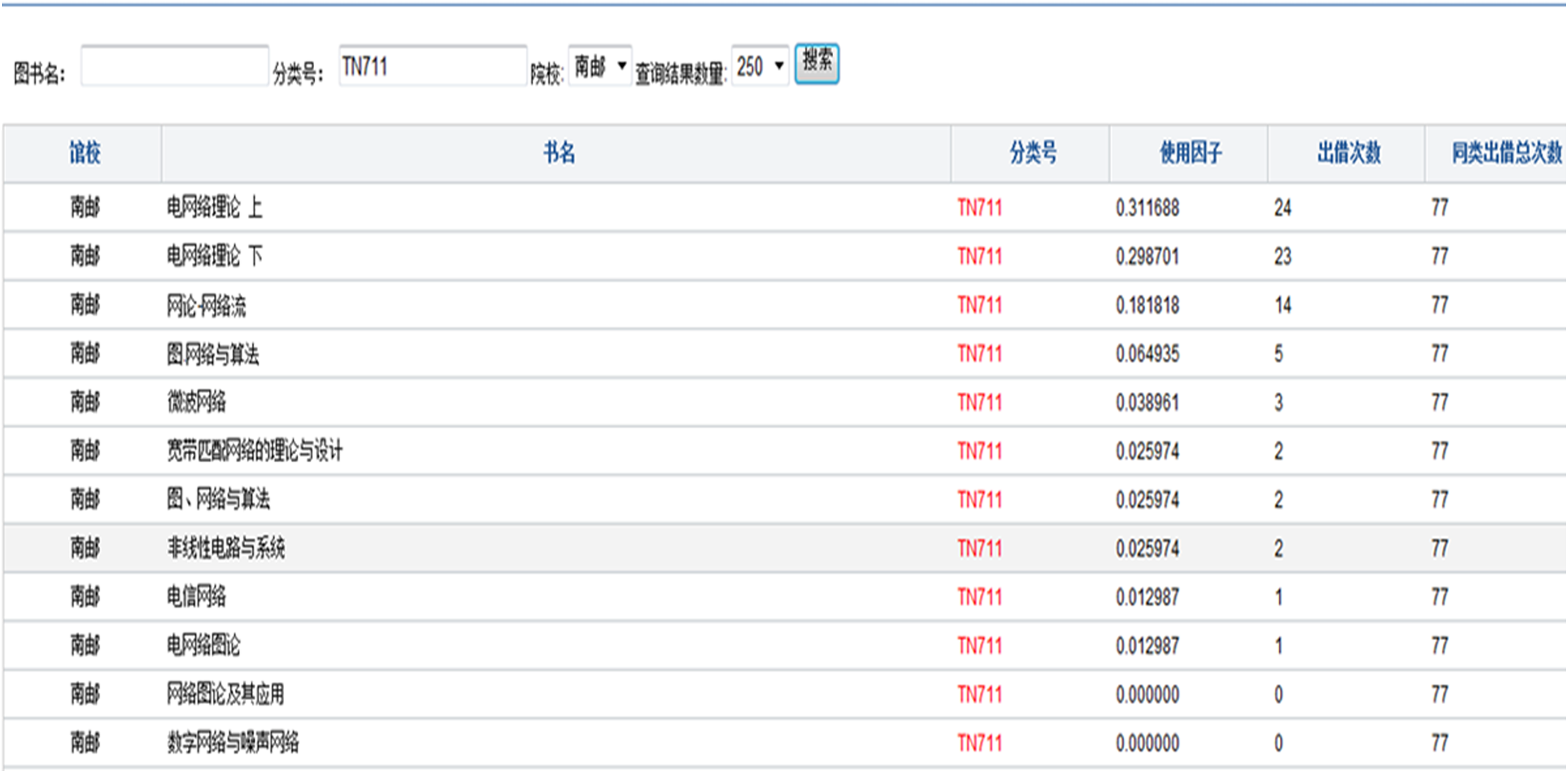

Supplement: Supplementary file 1 — Authors’ original file for figure 1 [file 40064_2012_203_MOESM1_ESM.tiff]

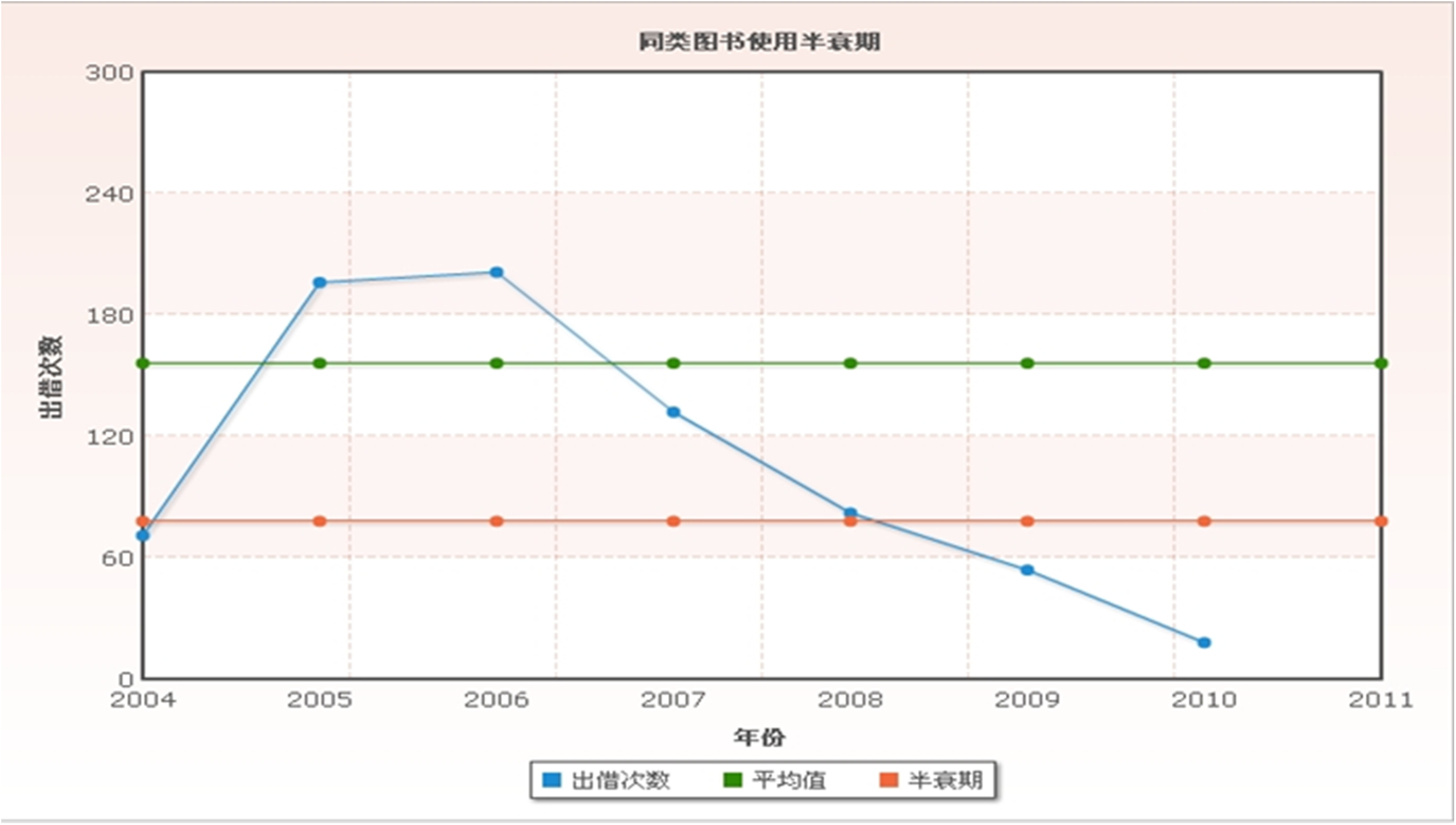

Supplement: Supplementary file 2 — Authors’ original file for figure 2 [file 40064_2012_203_MOESM2_ESM.tiff]

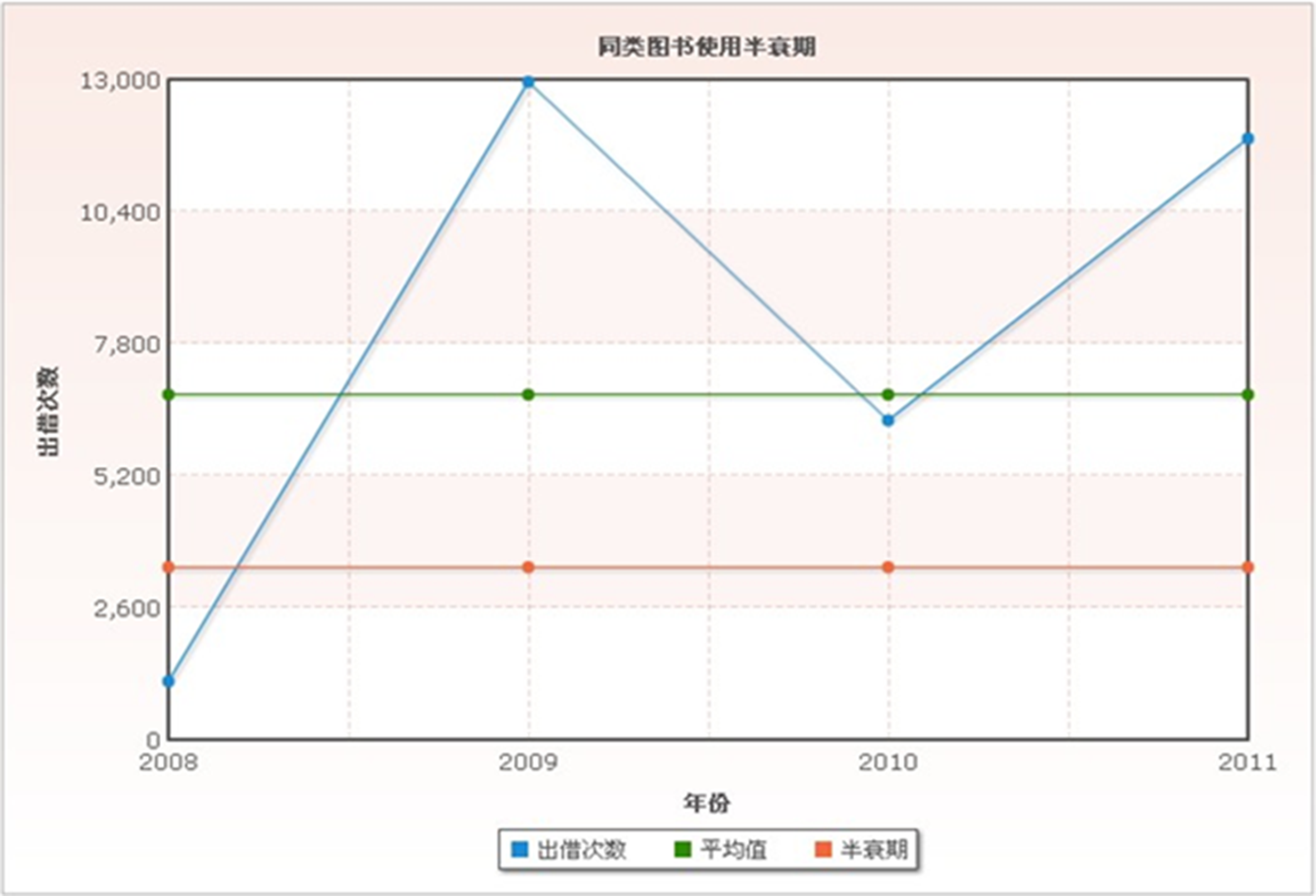

Supplement: Supplementary file 3 — Authors’ original file for figure 3 [file 40064_2012_203_MOESM3_ESM.tiff]
